# Educational interventions for knowledge on the disease, treatment adherence and control of diabetes mellitus

**DOI:** 10.1590/1518-8345.1648.2863

**Published:** 2017-04-20

**Authors:** Ana Laura Galhardo Figueira, Lilian Cristiane Gomes Villas Boas, Anna Claudia Martins Coelho, Maria Cristina Foss de Freitas, Ana Emilia Pace

**Affiliations:** 2 MSc, RN, Prefeitura Municipal de Lorena, Lorena, SP, Brazil.; 3 PhD, Professor, Centro Universitário da Fundação Educacional Guaxupé, Guaxupé, MG, Brazil. Professor, Faculdade Pitágoras de Poços de Caldas, Poços de Caldas, MG, Brazil.; 4 MSc, Professor, Faculdade de Taquaritinga, Taquaritinga, SP, Brazil.; 5 PhD, Associate Professor, Faculdade de Medicina de Ribeirão Preto, Universidade de São Paulo, Ribeirão Preto, SP, Brazil.; 6 PhD, Associate Professor, Escola de Enfermagem de Ribeirão Preto, WHO Collaborating Centre for Nursing Research Development, Universidade de São Paulo, Ribeirão Preto, SP, Brazil

**Keywords:** Diabetes Mellitus, Health Education, Knowledge, Medication Adherence, Patient Compliance

## Abstract

**Objective:**

to assess the effect of educational interventions for knowledge on the disease, medication treatment adherence and glycemic control of diabetes mellitus patients.

**Method:**

evaluation research with "before and after" design, developed in a sample of 82 type 2 diabetes mellitus patients. To collect the data, the Brazilian version of the Diabetes Knowledge Scale (DKN-A), the Measure of Adherence to Treatments and the electronic system at the place of study were used. The data were collected before and after the end of the educational interventions. The educational activities were developed within 12 months, mediated by the Diabetes Conversation Maps, using the Cognitive Social Theory to conduct the interventions.

**Results:**

the knowledge on the disease (p<0.001), the medication treatment (oral antidiabetics) (p=0.0318) and the glycated hemoglobin rates (p=0.0321) improved significantly.

**Conclusion:**

the educational interventions seem to have positively contributed to the participants' knowledge about diabetes mellitus, the medication treatment adherence and the glycated hemoglobin rates.

## Introduction

The morbidities associated with diabetes mellitus (DM) generally derive from the association between the long length of the disease and the bad glucose control^1^. After the establishment of the DM diagnosis, glicemic control is main objective of the treatment to prevent or retard its acute and chronic complications, promoting the quality of life and reducing the mortality^2^.

The treatment of type 2 DM (DM2) includes lifestyle changes, regular physical exercise and an appropriate diet. When the non-medication treatment does not achieve the expected results or adherence is unsatisfactory, the medication treatment is established, starting with oral antidiabetics (OADs) and, in certain associations, associated with insulin^2^. 

Treatment adherence is defined as the extent to which the person's behavior coincides with medical orientations in terms of medication use, diet, lifestyle changes or the adoption of behaviors to protect health^3^. The low adherence with the treatment of chronic conditions is an acknowledged problem around the world though. Adherence in developed countries is about 50% and can be even worse in developing countries^4^.

Treatment adherence demands that people take responsibility for their treatment and become active participants in a process that permits modulating the biological conditions through human behavior^5^. One of the factors that facilitate the acceptance and integration of the therapeutic regimen is people's knowledge about the disease^6^. 

In the context of care for DM patients, education to take care of the disease is an actions that permits the promotion/strengthening of the learning principles for healthy behavior^7^. 

Among the educational strategies that target DM patients, the Diabetes Conversation Maps are highlighted, which consist of playful and interactive illustrations and daily situations the people with this disease experience. The Diabetes Conversation Maps are a tool that engages people in the learning process to enable them to process the information in a concrete manner and use it in daily decision taking in DM management, as well as to stimulate the behavioral changes needed to control the disease and interact with the health professionals^8^. The tool should be used in group to permit the exchange of knowledge and experiences with other people in the same situation, thus facilitating learning^9^.

To guide and favor the learning process, the Diabetes Conversation Maps were conducted in accordance with the premises of the Social Cognitive Theory (SCT), also called Social Learning Theory^10^. Among the SCT constructs, Modeling is highlighted, a process that allows people to develop their behavioral and cultural standards, their beliefs and values, as a result of an ongoing interaction process with the environment. Therefore, the view of man in this theory is that of an individual inserted in social systems and, through the exchanges with this social midst, adaptation and change take place^11^.

In the search for effective educational strategies that promote behavioral changes, this study intended to assess the effect of educational interventions for knowledge on the disease, medication treatment adherence and glycemic control of diabetes mellitus patients.

## Method

Intervention study with single comparison group, developed at an outpatient clinic of a tertiary teaching hospital in the interior of the state of São Paulo, Brazil, between 2011 and 2013. The sample consisted of people medically diagnoses with DM2, male and female, with a minimum age of 40 years, under medication treatment using OAD and/or insulin, independently of the duration of the disease. This age limit was chosen due to the fact that DM2 is commonly diagnosed as from that age^2^.

People diagnosed with DM2 were excluded if they presented at least one of the following conditions: lesion or active ulcer in the lower limbs (LL), previous amputations at any LL level, under hemodialysis treatment and amaurosis, in a wheelchair and/or stretcher, sequelae of Cerebrovascular Accident (CVA), psychiatric diseases and others, difficulty to understand the instruments due to cultural factors, incapable of conversation, parallel participation in another educational group.

This study is linked to the matrix project entitled "Impact of a Care Program for Diabetes Mellitus Patients Centered on Educational Interventions and Social Support from Family", approved according to HCRP Process 9510/2010 and registered under Clinical Trial NCT01387633. This study sample was extracted from the matrix project, as described next. 

**Figure 1:**
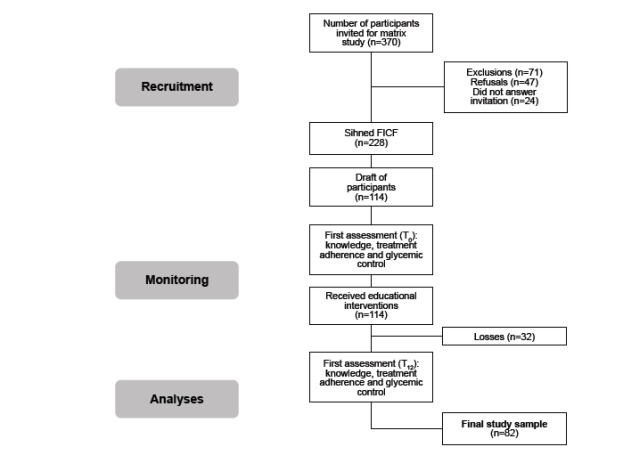
Research flowchart and sample constitution. Ribeirão Preto, SP, Brazil, 2013

In the baseline year for the recruitment, 1396 DM patients were monitored at the service where the study was developed. After the initial review, 485 patients complied with the inclusion/exclusion criteria. From this group, 370 people could be contacted to participate in the study. During to face-to-face recruitment process, the established criteria were again applied to the people who answered the invitation, showing that 71 presented at least one of the exclusion criteria, which was not mentioned in the patient history, leading to their exclusion. In addition, 47 people refused to participate in the study and 24 did not answer the invitation. Therefore, 228 people signed the Free and Informed Consent Form (FICF) and proceeded with the matrix study. Next, 114 were drafted to take part in the present study sample. Thirty-two of them quit due to the following reasons: 06 deaths, 03 exclusions (due to development of complications) and 23 dropouts. Therefore, 82 people concluded the study (Figure 1). 

For the sociodemographic characteristics, the following variables were considered: sex, age, education, marital status, origin and occupation, obtained through interviews with the participants and registered in an instrument structured for this purpose. The clinical variable analyzed was the length of the diagnosis. And the variables related to the treatment were: use of OADs, insulin, mean length of use, daily frequency of intake/application and class/type.

The knowledge on the disease was assessed by means of the Diabetes Knowledge Scale (DKN-A)^12^, translated and validated in a Brazilian population sample^13^, when it was called the Diabetes Knowledge Scale. To assess the treatment adherence, the "Measurement of Adherence to Drug Therapy in Diabetes Mellitus- Oral Antidiabetics" (MAT - OADs) and the "Measurement of Adherence to Drug Therapy in Diabetes Mellitus - Insulin Therapy" (MAT - Insulin)^14^ were used, tools taken from the document originally developed by Delgado and Lima^15^. These tools were used with the respective authors' authorization.

The DKN-A is a tool that assesses the general knowledge about DM. It consists of 15 multiple-choice items. Correct answers were scored "one" and incorrect answers "zero". Items 1 to 12 present a single correct answer and, for items 13, 14 and 15, there are two correct answers. The latter items are scored "one" when both alternatives marked are correct, and "0.5" if only one alternative is answered correctly. The total item scores determine the degree of knowledge and a final score of eight or higher has been considered as good knowledge^13^. 

In the test phase of the tools, five questions in the latter instrument were adjusted, as they were not clear to the DM patients who participated in that phase. The writing of questions 1, 2, 7, 9, 14 and 15 was adjusted. These adjustments are necessary, probably due to the fact that the tool was translated and validated in another region of Brazil, with particular characteristics in terms of language and eating habits (data not shown). 

The MAT-OAD and MAT-Insulin are Likert scales that consist of "seven" items, with six alternative answers, ranging from "always" to "never", corresponding to scores from "one" to "six", respectively. Adherence is determined by the global score, that is, the item scores are added up and divided by the total number of items (seven), with a range from one (1) to six (6). "Adherence" exists when the mean score ≥ 5, and "non adherence" when the mean score < 5^15^.

The glycemic control was assessed by means of the glycated hemoglobin (HbA1c) level, processed through High Performance Liquid Chromatography (HPLC), and the reference level < 7%, as established in the guidelines of the Brazilian Diabetes Society^2^. To collect this variable, all participants were specifically requested to undergo the test and, then, the results were collected from the internal electronic system of the place of study. 

In this study, four (4) maps were used that addressed the following themes: Map 1- "How do the body and the diabetes function", Map 2 - "Healthy eating and physical exercise", Map 3 - "Medication treatment and blood glucose monitoring" and Map 4 - "Reaching the targets with insulin". The educational sessions were conducted according to the protocol established for this purpose^16^ and based on the premises of the SCT, with a view to exploring the illustrations on the maps and present the participants' experiences and background knowledge to support the development of the group. 

Each participant attended six meetings, at mean intervals of three months. During the first meeting, the patients were invited, they signed the FICF and the first data were collected (before the educational interventions - T_0_). During the four successive meetings, the educational interventions took place, according to the themes proposed by means of the Diabetes Conversation Maps. During the sixth meeting, the second data were collected (after the educational interventions - T_12_). The duration of the educational interventions was 12 months.

The collected data were inserted in Excel through double data entry and processed electronically to validate the databases. The nominal variables were presented as absolute and relative frequencies and the numerical variables as means with standard deviation (SD) and median (with minima and maxima). The numerical data on the disease knowledge and treatment adherence scores and mean glycated hemoglobin levels were submitted to the Komolgorow-Smirnov and Levene tests to verify the normal distribution and homogeneity of the variances, respectively. 

To compare the scores between the two study times, that is, before and after the educational interventions, the paired Wilcoxon test was used. The statistical analyses were developed in R version 3.0.2. The differences were considered significant at *p* < 0.05. 

## Results

### Sociodemographic, clinical and treatment characteristics of the study sample

The final sample consisted of 82 people, 48 (58.5%) of whom were women and 34 (41.5%) men, with an average age of 60.43 (SD=8.38) years, and average education of 4.86 (SD=8.86) years. Concerning the sociodemographic characteristics, 59 (72%) were married/lived with a fixed partner, 44 (53.7%) came from the region of Ribeirão Preto and 44 (53.7%) were retired/pensioners. On average, the DM had been diagnosed for 15.38 (SD= 8.22) years.

What the treatment variables are concerned, 71 (86.6%) participants mentioned using OAD, with a mean length of use corresponding to 12.2 (SD=8.33) years, average daily consumption frequency of 2.5 times per day (SD=0.67) and the most frequently indicated therapeutic class were Biguanides in 46 (64.8%) cases. Sixty-eight (82.9%) participants mentioned insulin therapy, with a mean length of use equal to 8.3 (SD=5.83) years, average daily application frequency 2.2 times per day (SD=0.67) and the most used type in 35 (51.5%) cases was NPH mixed with Regular (R). 

### Knowledge about DM 

The Diabetes Knowledge Scale (DKN-A) score ranges from 0 to 15 points. The higher the score, the better the knowledge about the disease. The mean score increases between T_0_ and T_12_ for this variable at *p* < 0.05 (Table 1).

**Table 1:** Knowledge Assessment about DM (DKN-A) before (T_0_) and after (T_12_) the educational interventions. Ribeirão Preto, SP, Brazil, 2013

Dimensions	(N=82)		***p*** -value
	T**_0_**	T**_12_**	
Number of items	15		
Possible interval	0-15		
Minimum score	3	4	
Maximum score	14.5	15	
Mean (SD*)	9.44 (2.9)	10.8 (2.76)	< 0.001**^†^**
Median	10	11.5	

*SD: standard deviation

†Statistical significance (p-value < 0.05)

### Medication treatment adherence

Among the 71 participants who indicated using some class of OAD, 67 continued using the medication throughout the study, that is, before and after the educational interventions. Therefore, for this analysis, the 67 participants who answered the MAT - OAD at T_0_ and T_12_ were considered. For the 68 participants who indicated using some type of insulin, 67 continued using this medication throughout the study. The same number was considered for analysis, that is, the participants who answered the MAT - Insulin at T_0_ and T_12_.

The MAT score ranges from one to six. In the assessment of the medication treatment adherence (MAT - OADs), the means scores after the educational interventions (T_12_) were higher than the mean score at T_0_, with *p* < 0.05 (Table 2).

**Table 2:** Assessment of medication treatment adherence (MAT - OAD) before (T_0_) and after (T_12_) the educational interventions. Ribeirão Preto, SP, Brazil, 2013

Dimensions (N= 67)	MAT- OAD		
	T**_0_**	T**_12_**	***p*** -value
Number of items	7		
Possible interval	1-6		
Minimum score	2.43	2.14	
Maximum score	6.00	6.00	
Mean (SD*)	5.62 (0.60)	5.72 (0.52)	0.0318**^†^**
Median	5.71	5.86	

*SD: standard deviation

†Statistical significance (p-value < 0.05)

In the assessment of the medication treatment adherence (MAT - Insulin), a slight increase is observed in the mean score at T_12_, but without statistical significance (Table 3).

**Table 3:** Assessment of medication treatment adherence (MAT - Insulin), before (T_0_) and after (T_12_) the educational interventions. Ribeirão Preto, SP, Brazil, 2013

Dimensions (N=67)	MAT- INSULIN		
	T**_0_**	T**_12_**	***p*** -value
Number of items	7		
Possible interval	1-6		
Minimum score	3.71	4.43	
Maximum score	6.00	6.00	
Mean (SD*)	5.59 (0.47)	5.7 (0.29)	0.0588
Median	5.71	5.71	

*SD: standard deviation

### Glycemic control

The glycemic control results showed a reduction in the mean HbA1c from 9.3% (SD=1.89) and a median 8.95% (6.4-14.2) at T_0_ to 8.94% (SD=1.68) and a median 8.7% (5.7-13.2) at T_12,_ with *p*= 0.0321. 

## Discussion

This study showed that the educational intervention, mediated by the Diabetes Conversation Map and the premises of SCT, was effective to improve the knowledge on the disease, the medication treatment adherence and the glycemic control of DM2 patients.

Studies show that the DM2 patients lack knowledge on their disease^17^ ^-^ ^18^ and that this factor can affect the acceptance and integration of the therapeutic regimen^7^.

The World Health Organization^2^ presents education to chronic patients as an option to promote compliance, through motivation and personal training to use cognitive and behavioral strategies that facilitate adherence behaviors.

Different forms of educational activities have been used in DM patients and, to date, no universal model has been defined that can be standardized and acknowledged as effective for all patients^19^. Nevertheless, it is known that the success of these interventions depends on the person's ability to assume lifestyle changes, to maintain the recommended care, take initiative to identify, solve or seek help for the problems that emerge in the course of the disease^19^.

The applicability of the Diabetes Conversation Maps has been proven in studies developed in different countries. It is considered an effective and low-cost tool that permits interaction between the health professionals and the users in the construction of self-care^20^ ^-^ ^21^. Nevertheless, little is known about its effects on the knowledge, medication treatment adherence and glycemic control of DM2 patients. 

In a qualitative study among professionals working at Primary Health Care services in Belo Horizonte - Minas Gerais, aimed at analyzing the health professionals' view on the Diabetes Conversation Map, it was verified that, according to the professionals, this tool is a new strategy to construct self-care in diabetes and is appropriate to conduct educational practices^22^.

The use of innovative educational strategies like the Conversation Map has demonstrated its importance in care for DM patients, as it favors the professionals' improved knowledge, attitudes and skills to conduct the self-care practices, and mainly enables patients to understand their role in health care^21^ ^-^ ^22^. 

Associated with the appropriate choice of the tool to develop the educational interventions, the importance of adopting a theoretical framework to conduct the interventions is acknowledged. In that sense, the theoretical framework should permit the enhancement of the teaching-learning process, focusing on behavioral change for self-care^23^. The use of the SCT in this study favored the development of the educational interventions. 

The dialogue, experience reports and reflections on their own acts are an effective method to help DM patients embrace new life habits and develop and acquire self-care attitudes^24^. Therefore, in this study, an educational tool with a theoretical framework was used, which permitted the development of these attitudes. 

What the glycemic control is concerned, these findings are clinically relevant as, despite being superior to the control targets (> 7.0%) at T_12_, the mean glycated hemoglobin level found can be considered positive to slow down the chronic complications when considering the progressive nature of DM2^2^.

The clinical and metabolic improvement are post-intermediary results of the health education for DM2 patients^2^. A meta-analysis to assess the efficacy of DM education in the glycemic control of adult DM2 patients showed a mean reduction by 0.36% in the glycated hemoglobin levels^25^, similar to this research.

Although no correlation study was developed among the research variables, we can consider that the drop in the glycated hemoglobin levels results from the improved knowledge and treatment adherence the educative interventions provide.

As study limitations, the small sample size is highlighted, due to the exclusions, refusals and losses, which does not permit generalizations to the DM population. Clinical characteristics like the long length of the disease and the treatment (insulin use), as well as the characteristics of the place of study (tertiary care service) may have influenced the results. On the other hand, the lack of studies that used the Conversion Maps as an educational tool and the SCT as the theoretical structure made it difficult to establish comparisons with this study.

Despite the limitations mentioned, this study contributes to clinical nursing practices and appoints the need for further research, as no "gold standard" of health education for DM patients has been established thus far.

## Conclusion

These study results suggest that the educational intervention, mediated by the Diabetes Conversation Maps and conducted by means of the SCT, is an educational strategy that improves the knowledge about the disease, the treatment adherence and the glycemic control of DM2 patients. It can be executed at all health care levels and offers the DM patients means to develop skills in order to take care of the disease. Nevertheless, it requires professional training to conduct the educational activities in group.

Therefore, health professionals should use educational strategies mediated by tools that permit the patients' active participation in the teaching-learning process, with a view to achieving the behavioral changes needed to take care of the illness.
